# Detection of Hydrofluoric Acid by a SiO_2_ Sol-Gel Coating Fiber-Optic Probe Based on Reflection-Based Localized Surface Plasmon Resonance

**DOI:** 10.3390/s110201907

**Published:** 2011-02-01

**Authors:** I-Cherng Chen, Shiu-Shiung Lin, Tsao-Jen Lin, Je-Kang Du

**Affiliations:** 1 Micro Systems Technology Center, ITRI South, Industrial Technology Research Institute, Tainan 709, Taiwan; E-Mail: Eugenechen@itri.org.tw (I.-C.C.); 2 College of Dental Medicine, Kaohsiung Medical University, Kaohsiung 807, Taiwan;E-Mails: glasgow1993@yahoo.com (S.-S.L.); dujekang@ms31.hinet.net (J.-K.D.); 3 Chemical Engineering Department, National Chung-Cheng University, Chia-Yi, 621, Taiwan

**Keywords:** surface plasmon resonance, hydrofluoric acid, optical fiber, sol-gel, SiO_2_

## Abstract

A novel fiber-optic probe based on reflection-based localized surface plasmon resonance (LSPR) was developed to quantify the concentration of hydrofluoric acid (HF) in aqueous solutions. The LSPR sensor was constructed with a gold nanoparticle-modified PMMA fiber, integrated with a SiO_2_ sol-gel coating. This fiber-sensor was utilized to assess the relationship between HF concentration and SiO_2_ sol-gel layer etching reduction. The results demonstrated the LSPR sensor was capable of detecting HF-related erosion of hydrofluoric acid solutions of concentrations ranging from 1% to 5% using Relative RI Change Rates. The development of the LSPR sensor constitutes the basis of a detector with significant sensitivity for practical use in monitoring HF solution concentrations.

## 1. Introduction

Hydrofluoric acid (HF) is a contact poison and highly corrosive. It is used in certain specific chemical processes, such as wafer cleaning in the microelectronics industry [1], TFT-LCD manufacturing [2,3], and utilization for etching procedures on circuit boards and as a glass thinner. It is also used in dentistry to etch porcelain laminate veneers to produce micro-undercuts for bonding restoratives to fractured porcelain [4–6]. Nevertheless, the noxious characteristics of hydrofluoric acid can cause tremendous problems in the human body, such as severe burns and occupational poisoning. To effectively monitor online the concentration of HF is thus an enormously important and challenging task in chemical processing control which, in turn, will significantly affect product yields and environmental regulations.

Analytical methods currently employed to measure HF in an industrial environment include the infrared (IR) spectroscopic method [7,8], electrochemical methods [9], interferometry [10,11], acoustic wave sensors [12–15] and microcantilevers [16], *etc.* The IR spectroscopic method requires expensive and complex instrumentation and is therefore unsuitable for field analysis of multiple samples. Other acoustic wave devices such as surface transverse wave (STW) devices [12,13], LOVE-wave devices [14], and STW devices deposited on AT quartz coated by a fused silica layer [15] are excellent for real-time, in-field analysis of HF in the ppm level. Tang *et al.* applied a SiO_2_ microcantilever to detect HF at the femtomolal level [16]. However, the application of chemical processing control to online monitor the concentration of HF in weight-percent range of these systems has not been sufficiently developed.

Recently, a novel fiber-based sensor based on the particular optic properties of gold nanoparticles has been developed [17–19]. When an optical field is incident upon noble metal nanoparticles, absorption occurs if the optical frequency is resonant with the collective oscillation of the conduction electrons. This phenomenon is known as localized surface plasmon resonance (LSPR), and does not occur in bulk metals. The local environment of metal nanoparticles tends to significantly influence the resonance frequency and absorption of LSPR [20,21]. The light absorption level of gold nanoparticles is sensitive to both the refractive index (RI) of the surrounding solvent and the binding events of those functionalized nanoparticles.

The application of a SiO_2_ sol-gel coating LSPR for detecting HF is particularly feasible, as SiO_2_ tends to react uniquely with HF. The characteristics of HF to selectively react with SiO_2_ have been well reported [22–25]. The corresponding reaction schemes are as shown below:

(1) $$\text{At low concentration:}\;{\text{SiO}}_{2}+4\;\text{HF}\;\to \;{\text{SiF}}_{4}+2\;H_{2}O$$

(2) $$\text{At high concentration:}\;{\text{SiO}}_{2}+6\;\text{HF}\;\to \;H_{2}{\text{SiF}}_{6}+2\;H_{2}O$$

It seems promising to design a highly selective and sensitive detector for HF by utilizing the characteristics of SiO_2_ to selectively react with HF, but not with the other acids such as HCl or H_2_SO_4_, so its specificity to detect HF can be highly reliable. A novel SiO_2_ sol-gel coating LSPR sensor constructed with gold-nanoparticle modified optical fibers with high sensitivity for monitoring HF is therefore reported in this study. Instead of glass optical fiber, polyethylmethacrylate (PMMA) optical fiber [26] was used in this study to avoid the fibers being eroded by hydrofluoric acid during the detection process.

## 2. Materials and Methods

### 2.1. Reagents and materials

Plastic optical fibers (graded-index PMMA, Corning) were structured as the core and cladding with diameters of 400 and 430 μm, respectively. The chemicals (supplier) applied in this study included: *n*-hexadecyltrimethylammonium bromide (CTAB, Fluka), sodium borohydride (Lancaster), 3-(mercaptopropyl)trimethoxysilane (APTES, Acros), *N*-(2-mercaptopropionyl) glycine (MG, Fluka), cystamine dihydrochloride (Sigma), glutaraldehyde (Sigma), 1-ethyl-3-(3-dimethylaminopropyl)-carbo-diimide hydrochloride (EDC, Sigma), and *N*-hydroxysuccinimide (NHS, Sigma). All the aqueous solutions were prepared with purified water with a specific resistance of 18 MΩ cm generated by a Ropure ST water purification system (Barnstead).

### 2.2. Preparation of colloidal gold solutions

Hydrogen tetrachloroaurate solution (4.52 × 10^−4^ M) was prepared by mixing an aqueous solution of hydrogen tetracholoraurate (1.78 mL, 25.4 nM) with chloroform (8.22 mL) and an ethanol solution of CTAB (0.4 mL, 0.02 M). The mixed solution was thoroughly stirred for 10 minutes. Freshly prepared ethanolic NaBH_4_ solution (0.8 mL, 0.15 M) was added to the hydrogen tetrachloroaurate solution with vigorous stirring for 30 minutes. The ruby-colored organic phase was thereafter separated. Absorption spectra of the samples were investigated by utilizing a Hitachi U-2001 UV–VIS spectrophotometer. A Jeol transmission electron microscope (TEM) 1200EX was applied to observe the dispersed samples after they were dried on copper grids. Histograms derived from TEM image analysis showed that the mean diameter of the Au nanoparticles was 9.6 ± 2.3 nm.

### 2.3. Preparation of gold nanoparticle-modified PMMA optical fibers

The unclad portion (5 cm) of the PMMA optical fibers was cleaned and soaked in vials of NaOH (1M) for 24 hours. Subsequently these optical fibers were rinsed, dried, and submerged into the solution prepared by mixing APTES (0.0357 g), NHS (0.0295 g), EDC (0.0408 g) with 1% solution of APTES in EtOH contained within a 15-mL deionized water vials. The PMMA optical fibers were hence cleaned with ethanol and dried at 80 °C for ten minutes. As soon as its temperature dropped to the room temperature, the optic fibers were immediately immersed in *colloidal gold solutions* for 2 hours. They were thereafter withdrawn, rinsed, and soaked with deionized water for a day. The PMMA optical fibers modified with gold nanoparticles were thenceforth ready for this study once deionized water rinsing and drying were completed. TEM image analysis revealed that the thickness of the Au nanoparticles coating on the PMMA optical fiber was around 40 nm.

**Figure 1.**
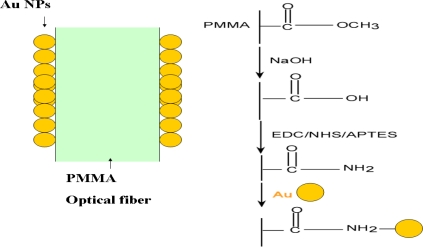
The diagram of gold nanoparticles immobilized on PMMA optical fiber.

The reaction mechanism of the immobilization and localization of the gold nanoparticles on PMMA optical fiber is shown schematically in Figure 1. The -COOCH_3_ functional groups on the PMMA surface tends to be hydrolyzed into -COOH groups as the PMMA was soaked in NaOH solution. The conversion of the -COOH functional groups into -CONH_2_ ones could be achieved by using EDC/NHS treatment with APTES. The -NH_2_ molecular structure is part of the terminal functional group in CONH_2_, which is modified on PMMA surface. The -NH_2_ structure inclines to bond with gold nanoparticles in water phase by electrostatic attraction between negative and CTAB-capped gold nanoparticles [27].

### 2.4. Preparation of PMMA optical fibers with sol-gel SiO_2_ coating

The synthesis of sol-gel solutions of SiO_2_ started with the preparation of the inorganic precursor sol from tetramethylorthosilicate (TMOS, 98%). The pH value during the preparation was adjusted by differential combination of HCl and NH_4_OH, which would act as catalysts. Since the structure of pore size and mechanical strength of bulk SiO_2_ were critically controlled by the molal ratio of H_2_O/TMOS during the sol-gel synthesis, this molal ratio (R value) was ranged from 4 to 15 in this study. The SiO_2_ coating process started with having sol-gel solutions dipped in the ice bath at 4 °C for 30 minutes, and afterwards it was placed in the environment at the room temperature for 150 minutes. The gold-modified PMMA optical fibers were then steadily inserted into the sol-gel; subsequently had them withdrawn at the speed of 3 cm per minute, and dried for 1 hour. These optic fibers were positioned into sol-gel filled cylindrical molds again at the room temperature for 12 hours, and thereafter the freeze-dried procedure was executed to obtain a layer of thick-film bulk coating. The coating thickness could be increased by repeating the molding procedure as needed.

### 2.5. HF detection

To enhance the reflection capacity of the LSPR fiber-optic probe, its distal ends were polished to form an optically smooth surface so that the reflection efficiency could be magnified. A silver film was then coated at the polished distal end. In this study, various lengths of unclad portions were used in order to obtain the optimal length for each type of gold-nanoparticle modified optical fibers (NM_Au_OFs). Their differential responses in terms of sensing capacity were compared in different refractive index (RI) media made up of sucrose solutions.

**Figure 2.**
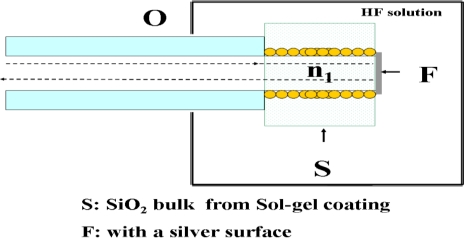
Schematic diagram of the reflection-based LSPR fiber-optic probe (**O**), with a silver surface (**F**), immersed in fluid vessel, containing a HF solution.

The LSPR sensor in this study was constructed with gold-modified nanoparticles on the unclad portion of PMMA fiber, which integrated with SiO_2_ sol-gel coating (Figure 2). A silver film [Figure 2(F)] was embedded to the terminal end of the unclad portion of the probe, and the light reflected by sensing material of SiO_2_ sol-gel coating was collected at the proximal end of the fiber probe.

For the assessment of sensing the sol-gel SiO_2_ coating LSPR fiber-optic probe was exposed to HF concentrations in a range from 0.25% to 8% for 30–120 minutes. At least five replicates were performed for each measurement. The sensitivity of the LSPR NM_Au_OFs sensor was defined as changes of the normalized intensity when measurement of a unit concentration of HF was performed.

### 2.6. Principle of the reflection-based LSPR fiber-optic sensor

When an optical field is incident upon noble metal nanoparticles, absorption will occur spontaneously if the optical frequency is resonant with the collective oscillation of the conduction electrons. This unique optical property makes LSPR sensing possible. However, the local environment of noble metal nanoparticles will significantly influence the resonance frequency and absorption of LSPR. Therefore, the absorption level of gold nanoparticles is highly sensitive to the refractive index (RI) of surrounding solvents as well as to the binding events of those functionalized nanoparticles.

The evanescent field absorption measurements of the fiber-optic were performed by comparing the light intensity of a sensor immersed in the analyzed HF solution at a testing condition (***I***) to the intensity of the sensor in a blank or a maximal value as a referring condition (***I_R_***). According to Nath and Chilkoti [28], the absorbance of the NM_Au_ changed in a solution of RI different from that of a blank. At an absorbance wavelength, the light attenuation can be described by Degrandpre and Burgess [29] as:

(3) $$-\;\text{log}\;(\frac{I}{I_{R}})=\text{log}\;(\frac{{\mathrm{NA}}_{R}^{2}}{{\mathrm{NA}}^{2}})+\eta_{P}\;\alpha \;L$$

The first term of Equation (3) describes that at a non-absorbing wavelength, the attenuation of absorbance signal results from the change in numerical aperture (***NA***), which is affected by a shift in critical angle for internal reflection [30]. In this term, the ***NA*** and ***NA_R_*** are given by:

(4) $${\mathrm{NA}}_{R}={\left({n_{1}}^{2}-{n_{2}}^{2}\right)}^{1/2}$$

(5) $$\mathrm{NA}={\left({n_{1}}^{2}-{n_{m}}^{2}\right)}^{1/2}$$

where ***n_1_*** is the core RI; ***n_2_*** is the cladding RI of a bare fiber (*i.e.*, RI of a blank), and ***n_m_*** is the effective cladding RI of Au nanoparticle-modified optical fiber (NM_Au_OF) immersed in an analyzed solution.

The second term of Equation (3) describes the attenuation caused by absorption of light in the cladding. It shows a pseudo-Beer’s-dependence of the absorbance on the absorptivity of NM_Au_, which will vary with NM_Au_OF length, density of gold nanoparticles on the fiber, and mean absorption cross-section of the gold nanoparticles. The power distribution ***η_p_*** is the fraction of total light intensity in the evanescent field; ***α*** is the absorptivity of NM_Au_ per unit length, and ***L*** is the fiber length. The power distribution can be calculated by the following equation [28]:

(6) $$\eta_{P}=(\frac{k\lambda }{2\pi r})\;\mathrm{NA}$$

where ***k*** is a constant; ***λ*** is the wavelength of light in the fiber core, and ***r*** is the fiber radius. According to Kennerly *et al.* [31], the absorptivity ***α*** of particle is defined as:

(7) α=log(1/1−Nσ)

where ***N*** is the number density of particles and ***σ*** is the cross area of a particle.

### 2.7. Instrumentation and measurements

**Figure 3.**
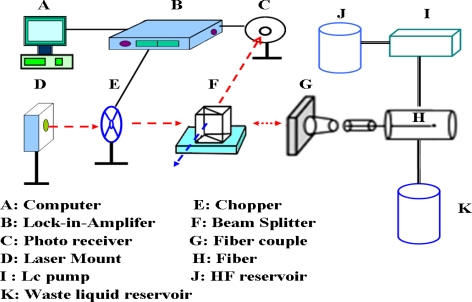
Schematic representation of the experimental setup applied to measurement with the self-assembled gold nanoparticle monolayer in the reflection-based LSPR fiber-optic probe on HF monitoring.

The configuration of the reflection-based LSPR sensing system for HF monitoring is shown in Figure 3. The system consisted of a laser (Hitachi HL6320G laser diode, 635 nm, 10 mW; Thorlabs LDC500 laser diode controller; Thorlabs TEC2000 temperature controller; Thorlabs TCLDM9 laser mount), a chopper (Stanford Research SR540), a beam splitter, a lock-in amplifier (Stanford Research SR830), a photoreceiver (Thorlabs PDA55), and the sensing unit maintained at 25 °C to stabilize the rate of SiO_2_ etching by the HF solutions.

## 3. Results and Discussion

### 3.1. Signal processing of LSPR sensing probe

**Figure 4.**
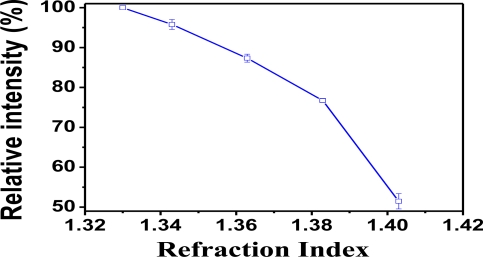
Plot of relative intensity *versus* refraction index (RI) of the medium obtained with Au-nanoparticle modified PMMA fiber in the configuration of SiO_2_/LSPR sensing probe.

To demonstrate HF detection via fiber optic probe, the reflection-based LSPR sensing system was fabricated with a Au-nanoparticle (NM_Au_) modified PMMA fiber, which integrated a SiO_2_ sol-gel coating. Figure 4 shows the plot of relative intensity *versus* refractive index (RI) of sucrose solution used to calibrate the RI measurments. This was obtained with NM_Au_ modified PMMA fiber in the SiO_2_/LSPR sensing probe configuration. The signal of the SiO_2_/LSPR sensing probe indicated a trend of decreasing relative intensity with increasing refractive index.

**Figure 5.**
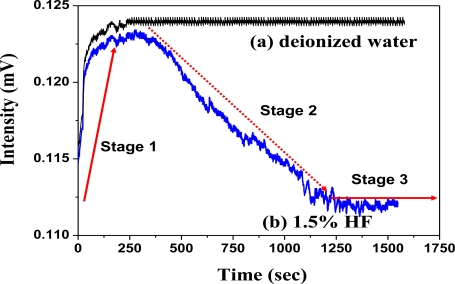
Dynamic responses of SiO_2_/LSPR sensing probe to (**a**) deionized water and (**b**) HF (1.5%), respectively (the bulk SiO_2_ had a diameter of 4.5 mm, at R = 10, in pH = 4 solution, after standing for 3 days).

**Figure 6.**
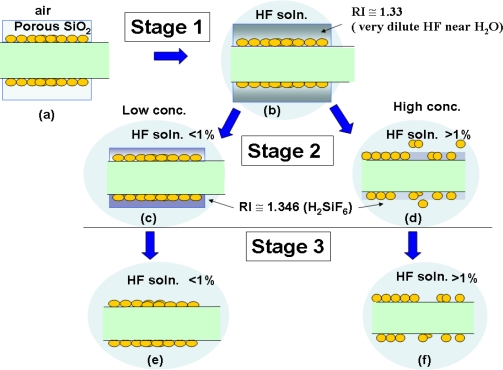
The stages of sensing mechanism of SiO_2_/LSPR sensing probe in the HFsolution. Stage-1: SiO_2_ porous layer was filled with solution (a∼b). Stage-2: SiO_2_ layer was eroded and produced H_2_SiF_6_ (H_2_SiF_6_ would deposit in the inner surface of porous layer: c∼d). Stage 3: SiO_2_ was completely eroded (e∼f). Yet the twofold effects of HF concentrations on SiO_2_ layer would induce different mechanisms to influence Relative RI Change Rates. One was the excessive accumulation of H_2_SiF_6_ in the porous structure (b→c→e) when HF concentration was less than 1%, and the other was the loss of attachment of NM_Au_ on PMMA optical fibers (b→d→f) when HF concentration was more than 1% (*please see the text*).

A comparison of the sensing responses between water as background and 1.5% HF solution was carried out [Figure 5(a); Figure 5(b)]. The sensing mechanism of the SiO_2_/LSPR sensing probe in HF solution could be sequentially categorized into three stages [Figure 5(b); Figure 6]. These 3-stage processes included:

1. stage-1: porous SiO_2_ layer was filled with HF solution (Figures 6a∼6b);
2. stage-2: SiO_2_ layer commenced to be eroded by HF, and thereafter produced H_2_SiF_6_, which would accumulate in the inner surface of SiO_2_ porous layer (Figures 6c∼6d);
3. stage-3: SiO_2_ layer was completely eroded (Figures 6e∼6f).

Its sensing characteristics in signal input [Figure 5(a); Figure 5(b)] demonstrated an increasing tendency when the LSPR sensing probe was initially inserted into the solution (Figure 6a). This phenomenon was due to the gradual water penetration into the porous layer of the SiO_2_ sol-gel coating (Figure 6b), which caused refractive index changes. The signal of the LSPR sensing probe when detecting deionized water tended to remain stable (Figure 5a). Nevertheless the signal gradually decreased in the HF solution as the SiO_2_ layer was eroded by HF (Figure 5b). This clearly implied the grounds for the declining signals as SiO_2_ layer was slowly corroded by HF. This induced the loss of NM_Au_ (Figure 6d), as well as reduced the amount of bonded NM_Au_, and consequently reduced in signal transduction of LSPR sensing probe (Figure 6f).

**Figure 7.**
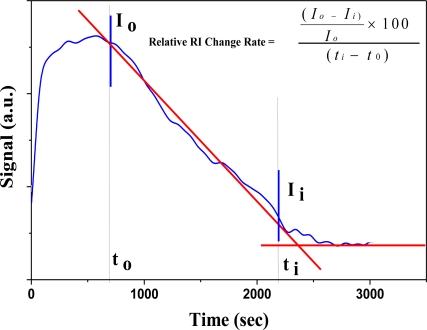
The calculation by numerical analysis of signals was represented as Relative RI Change Rate.

According to the dynamic responses of LSPR sensing probe to the HF solution (Figure 5b), the calculation by numerical analysis of the signal could represent Relative RI Change Rate as an indicator for measuring the concentration of HF in this study. Figure 7 shows the signal changes of LSPR sensing probe in the expression rate of the algorithm as Relative RI Change Rate. The signal change of LSPR sensing probe in HF solution was in the initial location of signal decrease. A tangent was drawn according the decreasing portion of the curve where there was a vertex intersection. This point was set as ***I_0_***, which corresponded to X axis of time ***t_0_***. Another tangent was performed in the region of signal stability after declination. There would be an intersection of two tangents; ***I_i_*** was set as the second intersection on declined signal curve plateau. It corresponded to X axis of time ***t_i_***. And Relative RI Change Rate was defined as:

(8) $$\text{Relative RI Change Rate}=\frac{\frac{\left(I_{0}-I_{i}\right)}{I_{0}}\times 100}{\left(t_{i}-t_{0}\right)}$$

### 3.2. LSPR sensing probe with SiO_2_ sol-gel coating

**Figure 8.**
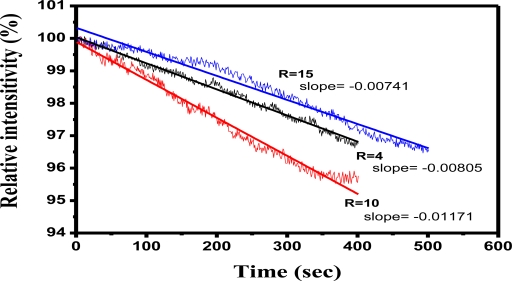
The comparative effect of the molal ratio of H_2_O/TMOS in the LSPR sensing probe with SiO_2_ sol-gel coating in detecting the HF solution (3.0%) at R = 4, R = 10, and R = 15, respectively (the formation of bulk SiO_2_ of 4.5 mm diameter, at pH = 4, after standing for 3 days).

The structure of the pore size and mechanical strength of bulk SiO_2_ were controlled by the molal ratio of H_2_O/TMOS in the sol-gel synthesis. Comparison of hydrolysis conditions for the formation of bulk SiO_2_ showed that if the H_2_O/TMOS molal ratio (R value) was low, this would imply a higher TMOS composition. Consequently bulk SiO_2_ would be hard, and brittle fractures tended to occur easily. In contrast, if R value was high, that would indicate a higher proportion of H_2_O. Therefore, the bulk SiO_2_ is relatively soft and it does not crack easily. Figure 8 shows the comparative effect of the molal ratio of H_2_O/TMOS in the LSPR sensing probe with SiO_2_ sol-gel coating in detecting 3.0% HF aqueous solution at R = 4, R = 10, and R = 15. HF etching caused the signal change in the slopes of the sensing curves at R = 4 (slope = −0.00805), R = 10 (slope = −0.01171), and R = 15 (slope = −0.00741), respectively. Observation of bulk SiO_2_ demonstrated that it tended to crack in the formation at R = 4, while the bulk material would contain much higher moisture at R = 15, and it inclined to soak easily in HF. The ideal formation of SiO_2_ bulk observed in this study was at R = 10.

**Figure 9.**
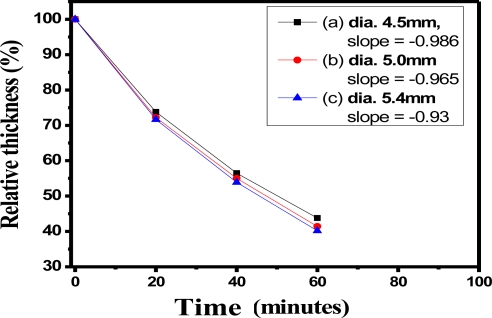
The curves of relative thickness *versus* time with SiO_2_ bulks in various diameters under the HF solution (1.0%), respectively (the formation of bulk SiO_2_ was at R = 10, pH = 4, after standing for 3 days).

Figure 9 reveals the curves of relative thickness *versus* time with bulk SiO_2_ of 4.5 mm diameter of (slope = −0.986), 5.0 mm (slope = −0.965), and 5.4 mm (slope = −0.930) in the HF solution (1.0%), respectively. For the same HF solution concentration, the slopes of bulk SiO_2_ samples with different relative thicknesses were similar to those resulting from erosion in this study, but thinner bulk SiO_2_ samples tended to be more sensitive.

**Figure 10.**
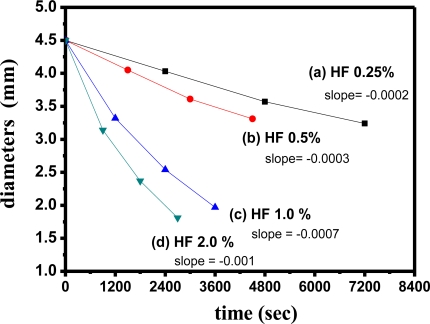
The curves of thickness change *versus* time, with 4.5-mm SiO_2_ bulk under the various HF concentrations of 0.25%, 0.5%, 1.0% and 2.0%, respectively (the formation of bulk SiO_2_ was at R = 10, at pH = 4, after standing for 3 days).

Figure 10 demonstrates the curves of thickness change *versus* time, with 4.5-mm bulk SiO_2_ for various HF concentrations of 0.25% (slope = −0.0002), 0.5% (slope = −0.0003), 1.0% (slope = −0.0007), and 2.0% (slope = −0.001), respectively. These thickness change *versus* time curves indicated that the slopes tended to decline much quicker as the concentration of HF increased. The comparisons among the curves of relative thickness *versus* time showed that the lower HF concentrations (≤0.5%) were almost linear lines, while those of the higher concentrations (≥1.0%) were closer to the second curves. Therefore, these results suggested the higher concentrations of HF (≥1.0%) would possess stronger penetration for bulk sol-gel SiO_2_ than those of lower concentrations (≤0.5%).

### 3.3. HF detection with the calculation of Relative RI Change Rate value of sol-gel SiO_2_/LSPR sensing probe

**Figure 11.**
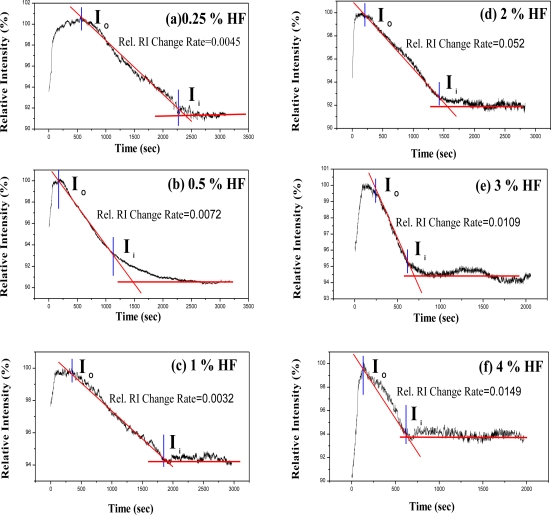
The comparison of relative RI change rates from calculation of the dynamic responses of SiO_2_/LSPR sensing probe to HF solutions in different concentrations (0.25%, 0.5%, 1.0%, 2.0%, 3.0% and 4.0%). The formation of SiO_2_ bulk of 4.5 mm in diameter with R = 10, pH = 4, after standing for 3 days.

**Figure 12.**
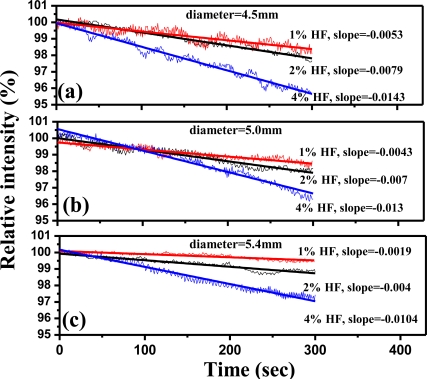
The curves of relative intensity *versus* time with various-diameter SiO_2_ bulks (4.5 mm, 5.0 mm, and 5.4 mm) in SiO_2_/LSPR sensing probe to the HF solutions with different concentrations (1.0%, 2.0%, and 4.0%). The formation of SiO_2_ bulk was at R = 10, pH = 4, after standing for 3 days.

In this study, signal processing of sol-gel coating SiO_2_/LSPR sensing probe to analyze HF solutions was represented as Relative RI Change Rates from calculation of the dynamic responses described in Figure 7 and Equation (8). The ideal condition of bulk sol-gel SiO_2_ formation was at R = 10, at pH = 4, after standing for 3 days. Figure 11 demonstrates the comparison of Relative RI Change Rates from calculating the dynamic responses of SiO_2_/LSPR sensing probe (diameter = 4.5 mm) to HF solutions of various concentrations (0.25%, 0.5%, 1.0%, 2.0%, 3.0% and 4.0%). Figure 12 shows the curves of relative intensity *versus* time with various-diameter bulk SiO_2_ samples (4.5 mm, 5.0 mm, and 5.4 mm) in SiO_2_/LSPR sensing probe under the various HF solutions of 1.0%, 2.0%, and 4.0%, respectively. For identical HF concentrations, the slopes of bulk SiO_2_ with relative intensity change were similar to those of erosion in this study (Figure 9), and the sensing probe with the smaller thickness tended to be more sensitive. These curves of intensity change *versus* time indicated the slopes would decline much quicker as the concentration of HF increased.

**Figure 13.**
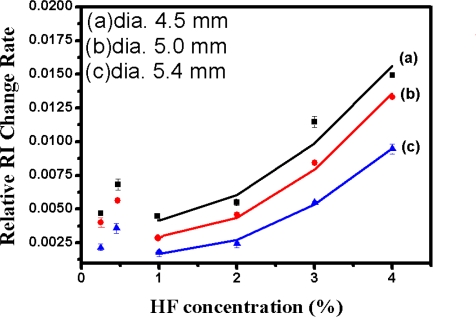
The comparison of relative RI change rates of SiO_2_/LSPR sensing probe with various-diameter SiO_2_ bulks (4.5 mm, 5.0 mm, and 5.4 mm) to detect the HF solutions in different concentrations (0.25%, 0.5%, 1.0%, 2.0%, 3.0%, and 4.0%). The formation of SiO_2_ bulk was at R = 10, pH = 4, after standing for 3 days.

Statistical data from repeated measurements (*n = 3*) obtained in the same Relative RI Change Rates from calculating the dynamic responses of SiO_2_/LSPR sensing probes of 4.5 mm, 5.0 mm, and 5.4 mm in diameter was tabulated in Table 1. The data was also plotted in Figure 13 to represent the curves of Relative RI Change Rates *versus* concentrations of HF detected by SiO_2_/LSPR sensing probe with various-diameter bulk SiO_2_ samples for various HF concentrations of 0.25%, 0.5%, 1.0%, 2.0%, 3.0%, and 4.0%, respectively. Figure 13 indicated that Relative RI Change Rates declined as HF concentration decreased between the observed ranges from 4% to 1%, but that it increased in the 0.5% HF solution, and subsequently it declined again when the HF concentration was 0.25%.

These interactions between Relative RI change Rates and HF concentration-curve for various-diameter bulk SiO_2_ samples can be proposed as below. The calculation of Relative RI Change Rates from the dynamic responses of SiO_2_/LSPR sensing probe on HF detection indicate the decrease of signal intensity was graphically demonstrated in stage-2 of Figure 5. The original refractive index of the diluted HF solution was 1.33. Initially, the SiO_2_ bulk was profoundly penetrated by HF, and produced SiF_4_ as well as H_2_O. The large amounts of these products present would therefore generate silicon fluoride acid (H_2_SiF_6_) with refractive index 1.3460. Logically, the eroded SiO_2_ by HF was very likely to generate more H_2_SiF_6_ with the larger refractive index, and resulted in speedy signal decline of the LSPR sensing probe.

Nevertheless, the reasons of signal shifts of SiO_2_/LSPR sensing probe in different concentrations of HF solution were twofold. One (***a-effect***) was the accumulation of the produced H_2_SiF_6_ would increase refractive index of medium, which tended to diminish the relative intensity (Figure 4). The other (***b-effect***) was the lost attachment of NMAu on PMMA optical fibers, which occurred during the SiO_2_erosive process. This tended to decrease the SPR functions. These twofold effects produced by HF to corrode SiO_2_ may possess two differential mechanisms under the different concentrations of HF solution.

The surface reaction mechanism of SiO_2_/LSPR sensing probes to HF solution with concentrations ranged from 4% to 1% was demonstrated in Figures 6d and 6f. Although the reaction would produce significant amounts of H_2_SiF_6_, the porous structure of SiO_2_ had been destroyed and therefore the amount of H_2_SiF_6_ existing on the SiO_2_ structure would be minimal. The major contribution to signal change in this condition would be ***(b)-effect***, *i.e.*, the loss of NMAu and impairment of the SPR functions due to the erosion of SiO_2_.

However, the change of Relative RI Change Rates in the low concentrations of HF solution (≤0.5%) was mainly contributed by large amounts of accumulated H_2_SiF_6_ in the porous structure (Figure 6c; Figure 6e). The erosive process initially only produced the infiltration of porous SiO_2_ bulk and slightly surface etching (Figure 6b). It meant that ***(a)-effect*** brought about by the excessive deposition of H_2_SiF_6_ would increase the refractive index and reduce the relative intensity under these circumstances, rather than the enormous loss of attached NM_Au_ on PMMA optical fibers. The same situation of H_2_SiF_6_ deposition would also occur in HF solution of 0.25%. Yet the amount of produced H_2_SiF_6_ remained in the porous structure would be less than that in 0.5% HF solution.

In this study, SiO_2_ bulks of SiO_2_/LSPR sensing probe were 4.5 mm, 5.0 mm and 5.4 mm in diameter with the formation at R = 10, at pH = 4, after for 3 days. It was suitable to detect the HF solution with concentrations ranging from 1 to 5%. However, SiO_2_/LSPR sensing probe will be quickly eroded with higher concentrations of HF (>8%), and it will be more difficult to reproduce the detecting capability of SiO_2_/LSPR sensing probes. As to detecting HF solutions o low concentration (<1.0%), it requires further investigation to design the appropriate SiO_2_ film, so that the precise sensing in this field can be achieved.

## 4. Conclusions

A novel fiber-optic probe with reflection-based localized surface plasmon resonance (LSPR) was developed to quantitatively assess the concentration of hydrofluoric acid (HF) solution. The LSPR sensor was fabricated with a gold-nanoparticle modified PMMA fiber, integrated with a SiO_2_ sol-gel coating. This LSPR sensing probe was applied to evaluate the relationship between HF concentrations and SiO_2_ sol-gel layer etching reduction. The results demonstrated the LSPR sensor was properly designed to detect HF-related erosion rate with Relative RI Change Rates ranged from 1% to 5% of hydrofluoric acid solution and thus constitutes the basis of its use as a detector applicable to monitoring HF solution. Further investigations are required in order to produce reliable fully functional sensors for either higher (>8%) or lower (<1%) concentrations of HF solution.

## Figures and Tables

**Table 1.** Comparisons of Relative RI Change Rates from calculation of the dynamic responses of SiO_2_/LSPR sensing probes in HF solutions (The replicated measurements = 3).

**The concentrations of HF solution**	**The diameters of sol-gel SiO_2_ of LSPR sensing probes**		

	4.5 mm	5.4 mm	5.4 mm

	Relative RI change rates		

0.25%	0.0047 ± 0.00016	0.0040 ± 0.00036	0.0022 ± 0.00025
0.5%	0.0074 ± 0.00017	0.0060 ± 0.00025	0.0040 ± 0.00020
1%	0.0033 ± 0.00008	0.0022 ± 0.00012	0.0019 ± 0.00012
2%	0.0055 ± 0.00022	0.0046 ± 0.00021	0.0024 ± 0.00012
3%	0.0114 ± 0.00040	0.0084 ± 0.00012	0.0055 ± 0.00008
4%	0.0149 ± 0.00012	0.0133 ± 0.0008	0.0095 ± 0.00037
